# Routine Versus Deferred Percutaneous Coronary Intervention in Patients Undergoing Transcatheter Aortic Valve Replacement: A Meta-analysis With Reconstructed Individual Patient Data

**DOI:** 10.1016/j.jscai.2026.105566

**Published:** 2026-08-04

**Authors:** Mahmoud Balata, Ahmed Samy Badran, Mohamed Ibrahim Gbreel, Marwa Hassan, Islam Ebeid, Abdelhakim A. Abdelrahman, Daniel Robinson, Jonas Bodanowitz, Stephan Valenta, Christin Valenta, Plamen Kochev, Hüseyin Ince, Jasmin Ortak

**Affiliations:** a Department of Cardiology, University Hospital of Rostock, Rostock, Germany; b Faculty of Medicine, Ain Shams University, Cairo, Egypt; c Department of Cardiology, Ahmed Maher Teaching Hospital, Cairo, Egypt; d Theodor Bilharz Research Institute, Giza, Egypt; e Department of Cardiology, National Institute of Diabetes and Endocrinology, Cairo, Egypt; f Emergency Department, King's College Hospital, London, United Kingdom

**Keywords:** aortic stenosis, coronary artery disease, meta-analysis, percutaneous coronary intervention, revascularization timing, transcatheter aortic valve replacement

## Abstract

**Background:**

The optimal management of concomitant coronary artery disease in patients undergoing transcatheter aortic valve replacement (TAVR) remains controversial. Whether routine percutaneous coronary intervention (PCI) before TAVR provides clinical benefit compared to a strategy of deferral is unclear.

**Methods:**

We performed a meta-analysis of randomized controlled trials (RCT) comparing routine PCI before TAVR with a strategy of PCI deferral. The primary end points were a composite of major adverse cardiovascular and cerebrovascular events (MACCE) and all-cause mortality. Secondary end points included myocardial infarction, stroke, revascularization, and major bleeding. We applied a random-effects model to calculate hazard ratios (HR) and 95% CI. We also performed a pooled time-to-event analysis using reconstructed individual patient data.

**Results:**

We included 3 RCT (ACTIVATION, NOTION-3, and PRO-TAVI) comprising a total of 1156 patients. The pooled cohort had a median age exceeding 81 years and exhibited low anatomic coronary complexity (median SYNTAX score ≤10). Routine PCI did not significantly reduce the risk of MACCE (HR, 0.84; 95% CI, 0.62-1.13; *P* = .25) or all-cause mortality (HR, 0.88; 95% CI, 0.67-1.17; *P* = .38). Pooled Kaplan-Meier analysis confirmed no difference in all-cause mortality over 48 months (HR, 0.89; 95% CI, 0.68-1.17; *P* = .41). Although routine PCI was associated with a significant reduction in any revascularization (HR, 0.24; 95% CI, 0.06-0.89; *P* = .03) and urgent revascularization (HR, 0.33; 95% CI, 0.12-0.87; *P* = .02), it was associated with increased bleeding events (HR, 1.96; 95% CI, 1.28-3.00; *P* < .01). No significant differences were observed regarding myocardial infarction or stroke.

**Conclusions:**

Routine PCI before TAVR did not reduce mortality or MACCE compared to a deferral strategy. Although routine PCI lowered the risk of subsequent urgent revascularization, it significantly increased the risk of major bleeding.

## Introduction

Severe aortic stenosis (AS) is the most common valvular heart disease in older adults and carries a poor prognosis once symptoms develop. Transcatheter aortic valve replacement (TAVR) has transformed the management of AS, with continued expansion expected as indications broaden.^1^^,^^2^ Coronary artery disease (CAD) frequently coexists with severe AS. CAD affects approximately one-half of patients undergoing TAVR, owing to shared risk factors and pathobiology.^3^^,^^4^ In surgical aortic valve replacement, concomitant coronary artery bypass grafting is routinely recommended for significant coronary disease.^5^ Whether routine percutaneous coronary intervention (PCI) should, similarly, accompany TAVR remains uncertain. Although PCI may reduce ischemic burden and subsequent coronary events, it increases procedural complexity and often necessitates dual antiplatelet therapy in an older population already vulnerable to bleeding.^6^

Current European (European Society of Cardiology/European Association for Cardio-Thoracic Surgery) and American (American College of Cardiology/American Heart Association) guidelines started to support a selective approach and recommend PCI for prognostically important lesions rather than routine revascularization in all patients undergoing TAVR.^1^^,^^7^ However, practice remains heterogeneous because the available evidence has been limited and conflicting. Observational studies showed no survival advantage with routine PCI.^4^ The ACTIVATION trial found no improvement in clinical outcomes and identified increased bleeding with pre-TAVR PCI.^8^ More recently, the NOTION-3 trial suggested fewer ischemic events with routine revascularization, albeit at the expense of greater bleeding.^9^ In contrast, the PRO-TAVI trial demonstrated that PCI deferral was noninferior to routine PCI while reducing major bleeding.^10^

Thus, the optimal revascularization strategy in patients undergoing TAVR remains unresolved. Individual trials have differed. Therefore, we performed a meta-analysis of randomized controlled trials (RCT) comparing routine versus deferred PCI in patients undergoing TAVR.

## Methods

This study was conducted in adherence to the Cochrane Handbook for Systematic Reviews of Interventions and is reported in accordance with the Preferred Reporting Items for Systematic Reviews and Meta-Analyses (PRISMA) 2020 statement.^11^^,^^12^ The review protocol was registered on PROSPERO (CRD420261358039).

### Search strategy and screening

A systematic search of MEDLINE (via PubMed), the Cochrane Central Register of Controlled Trials, Web of Science Core Collection, and Scopus from database inception to April 1, 2026. The search strategy used a combination of controlled vocabulary: (1) TAVI/TAVR and related device terminology; (2) coronary artery disease, PCI, and revascularization strategy terminology; and (3) a broad randomized trial identifier. Within each block, controlled vocabulary (MeSH for PubMed) was combined with free-text terms using the Boolean operator OR; the 3 blocks were then intersected using AND. The complete strategy for each database is available in Supplemental Table S1. ClinicalTrials.gov was queried for ongoing trials, reference lists of all retrieved full-text articles, and the late-breaking clinical trial proceedings of the American College of Cardiology (ACC 2026) were screened to capture recent data. No restrictions were applied with respect to language, country of origin, or publication date.

All retrieved records were imported into EndNote and duplicated. Two reviewers (A.S.B., M.I.G.) independently screened titles and abstracts, followed by a full-text assessment of eligible articles. A third author (M.B.) resolved conflicts.

### Eligibility criteria

We defined our inclusion criteria using the PICOS framework. We restricted inclusion to RCT; observational cohorts, retrospective registries, and nonrandomized study designs were excluded. Eligible trials enrolled adult patients with severe, symptomatic AS and concomitant significant CAD who were accepted for TAVR following multidisciplinary Heart Team evaluation. “Significant CAD” was defined by either angiographic severity (eg, ≥70% or ≥90% diameter stenosis in a major epicardial vessel) or physiological significance (eg, fractional flow reserve [FFR] ≤0.80). The intervention was defined as a strategy of routine PCI, performed either prior to or concomitantly with the TAVR procedure. The comparator was a deferred or conservative strategy, wherein patients proceeded directly to TAVR without prophylactic coronary stenting, and PCI was strictly reserved as a subsequent procedure.

### Data extraction and end points

Two investigators (A.S.B., M.I.G.) independently extracted trial-level data using standardized forms. We extracted trial-level characteristics, including study design, enrollment period, follow-up duration, specific inclusion criteria, and primary composite end point definitions. Baseline clinical variables included patient age, sex distribution, surgical risk estimates (Society of Thoracic Surgeons Predicted Risk of Mortality [STS PROM] and EuroSCORE II), and prevalent comorbidities (diabetes mellitus, prior myocardial infarction [MI], prior coronary artery bypass grafting, and atrial fibrillation). We captured baseline symptom burden using the New York Heart Association functional class and the presence of clinical angina. Extracted echocardiographic parameters included left ventricular ejection fraction, mean transvalvular pressure gradient, and aortic valve area. Angiographic and procedural variables comprised the trial-defined threshold for significant coronary disease, utilization of invasive physiology, baseline Synergy between Percutaneous Coronary Intervention with TAXUS and Cardiac Surgery (SYNTAX) scores, PVI timing, stent type, valve type, and access route. Discrepancies were resolved through discussion or adjudication by a third investigator (M.B.).

The primary end points were major adverse cardiovascular and cerebrovascular events (MACCE), as per trial definition, all-cause mortality, and cardiovascular death. Secondary end points included the following: MI, stroke, bleeding, any or urgent coronary revascularization, and acute kidney injury. Outcome definitions were harmonized across trials to the extent permitted by available data, and any deviations are reported transparently.

### Quality Assessment

The quality of included studies was independently evaluated by 2 investigators (M.I.G. and A.S.B.). Disagreements were resolved by consensus or by a third author (M.B.). The Cochrane Risk of Bias 2.0 (RoB 2) tool^13^ was used for RCT.

### Statistical analysis

All analyses were performed using RStudio version 4.4.3 (R Foundation for Statistical Computing). For time-to-event dichotomous outcomes, we calculated pooled hazard ratios (HR) with 95% CI. Where the longest follow-up HRs were reported, the log HR and its standard error were pooled directly. Given the anticipated clinical and methodological diversity across trials (eg, PCI guidance strategies, valve types), a random-effects model was used. Statistical significance was defined as *P* <.05. Heterogeneity was assessed using the I^2^ statistic, with thresholds of 0% to 49%, 50% to 75%, and >75% interpreted as low, moderate, and high heterogeneity, respectively. Sensitivity analyses were performed by excluding each study sequentially (leave-one-out approach). This methodological step assesses the robustness of the pooled estimates and identifies whether a single trial exerted disproportionate influence on the overall findings. Given the number of eligible RCT, formal evaluation of publication bias using Egger's test or funnel plots was considered underpowered. We reconstructed individual patient data (IPD) from published Kaplan-Meier (KM) survival curves for all-cause mortality. High-resolution digital extraction of KM coordinates was performed using WebPlotDigitizer. These digitized survival data, together with the total number of patients, events, and number-at-risk tables at specified time points, were processed using the iterative algorithm described by Guyot et al (2012)^14^ via the IPDfromKM package in R. The reconstructed IPD was additionally cross-validated by comparing the reconstructed data with the reported data. Cox proportional hazards (PH) models were then fitted to the reconstructed IPD to estimate study-level HR. Schoenfeld residuals were used to test the PH assumption.

## Results

### Study selection

Our systematic literature search identified 3203 records across databases. We removed duplicates and screened 2114 titles and abstracts for relevance. We retrieved 25 full-text articles for full-text evaluation. The final quantitative synthesis included 3 studies^8^, ^9^, ^10^ (Supplemental Figure S1).

### Characteristics of included studies

Three multicenter RCT (ACTIVATION, NOTION-3, PRO-TAVI) comprised a total of 1156 patients.^8^, ^9^, ^10^ The ACTIVATION and PRO-TAVI trials report clinical outcomes at 12 months. The NOTION-3 trial reports outcomes at a median of 24 months (Supplemental Table S2). Baseline clinical characteristics were well balanced across the deferred and routine PCI arms (Table 1).^8^, ^9^, ^10^ The pooled cohort represents an older demographic with a median age exceeding 81 years in all studies. Surgical risk was consistently categorized as low to intermediate. The median STS PROM score ranged from 3.0% in the NOTION-3 cohort to 4.4% in the ACTIVATION cohort. Coronary anatomic complexity was uniformly low among the evaluated patients (Table 1). The NOTION-3 and PRO-TAVI trials reported median baseline SYNTAX scores of 9.0 and 10.0, respectively. The individual trials used heterogeneous definitions for significant CAD. The ACTIVATION trial relied exclusively on an anatomic threshold of 70% diameter stenosis, whereas the NOTION-3 and PRO-TAVI trials incorporated invasive physiological assessments. Procedural approaches for both the coronary and valvular interventions evolved across the study periods. Bare-metal stents were implanted in 17.6% of the PCI arm in the ACTIVATION trial. The contemporary NOTION-3 and PRO-TAVI trials used drug-eluting stents exclusively. Balloon-expandable platforms were used in 88.8% of the ACTIVATION cohort compared to roughly half of the patients in the 2 newer trials. Using the RoB 2 tool, the 3 included trials were rated as low risk of bias (Supplemental Figure S2).

**Table 1:** Baseline clinical, angiographic, and procedural characteristics of the included trials

Trial	ACTIVATION^8^		NOTION-3^9^		PRO-TAVI^10^	
	Deferral (n = 116)	PCI (n = 119)	Deferral (n = 228)	PCI (n = 227)	Deferral (n = 233)	PCI (n = 233)
Clinical characteristics						
Age, y	84.3 ± 5.0^a^	83.6 ± 5.0^a^	81 (78-85)	82 (78-85)	81 (77-84)	81 (78-84)
Male sex	75 (64.7)	69 (58.0)	153 (67.1)^b^	154 (67.8)^b^	149 (64.0)	151 (65.0)
STS-PROM, %	4.4	4.4	3	3	3.1	3.1
Diabetes mellitus	27 (23.3)	35 (29.4)	61 (27.0)	59 (26.0)	75 (32.0)	75 (32.0)
Prior myocardial infarction	26 (22.8)	14 (11.8)	24 (11.0)	14 (6.0)	46 (20.0)	57 (24.0)
Prior CABG	11 (9.6)	8 (6.7)	10 (4.0)	7 (3.0)	13 (6.0)	19 (8.0)
Atrial fibrillation	N/R	N/R	74 (32.0)	81 (36.0)	80 (34.0)	82 (35.0)
NYHA class III or IV	65 (56.0)^c^	71 (59.7)^c^	95 (41.7)^c^	105 (46.3)^c^	123 (53.0)	116 (50.0)
Clinical angina present	37 (31.9)^d^	38 (31.9)^d^	N/R	N/R	85 (36.0)	103 (44.0)
Angiographic and procedural						
Angiographic CAD criteria	≥70% stenosis		≥90% or FFR ≤0.80		≥70% stenosis or physiology	
Invasive physiology used	0 (0)	0 (0)	N/R (155 lesions)	N/R (167 lesions)	32 (14.0)	33 (14.0)
SYNTAX score, median	N/R	N/R	9	9	10	10
Transfemoral TAVR access	86 (80.4)^e^	98 (89.0)^e^	N/R	N/R	214 (93.0)^e^	214 (93.0)^e^
Balloon-expandable valve	95 (88.8)^e^	103 (93.6)^e^	95 (42.0)	90 (40.0)	125 (55.0)^e^	117 (51.0)^e^
Timing of PCI (before TAVR), %	N/A	100.0^f^	N/A	74.0^g^	N/A	93.0^g^
Bare-metal stent used	N/A	21 (17.6)	N/A	0 (0)	N/A	0 (0)
Complete revascularization, %	N/R	N/R	N/A	89	N/R	N/R (residual SYNTAX 0)

Values are presented as mean ± SD, median (IQR), or n (%), unless otherwise specified.

CABG, coronary artery bypass grafting; CAD, coronary artery disease; FFR, fractional flow reserve; N/A, not applicable; N/R, not reported; NYHA, New York Heart Association; PCI, percutaneous coronary intervention; STS-PROM, Society of Thoracic Surgeons Predicted Risk of Mortality; SYNTAX, Synergy between Percutaneous Coronary Intervention with TAXUS and Cardiac Surgery; TAVR, transcatheter aortic valve replacement.

a The ACTIVATION trial reported age as mean ± standard deviation.

b The NOTION-3 trial reported female sex; male sex was mathematically derived for harmonization (100% – female%).

c Derived by summing the raw frequencies of NYHA class III and NYHA class IV reported in the primary texts.

d Derived by subtracting patients with CCS class 0 from the total cohort to determine the presence of any angina.

e Denominator is based on the number of patients who actually underwent the TAVR procedure, which varied slightly from the total randomized population in the ACTIVATION and PRO-TAVI trials because of dropouts or procedural cancellation.

f The ACTIVATION trial protocol mandated PCI prior to TAVR.

g The NOTION-3 and PRO-TAVI trials allowed PCI concomitantly with or shortly after TAVR; percentages reflect the proportion of the PCI arm that underwent the procedure strictly before the TAVR intervention.

### Primary clinical outcomes

Compared with the deferral strategy, routine PCI did not significantly reduce the risk of MACCE (HR, 0.84; 95% CI, 0.62-1.13; *P* = .25), with low-to-moderate between-study heterogeneity (I^2^ = 37.2%) (Figure 1A).^8^, ^9^, ^10^ Trials’ definitions of MACCE are presented in Supplemental Table S3. Similarly, there was no significant difference observed for all-cause mortality across the 3 included trials (HR, 0.88; 95% CI, 0.67-1.17; *P* = .38; I^2^ = 0.0%) (Figure 1B).^8^, ^9^, ^10^ Analysis of cardiovascular death also demonstrated no significant difference between the treatment groups (HR, 0.77; 95% CI, 0.49-1.19; *P* = .23), with no statistical heterogeneity (I^2^ = 0.0%) (Figure 1C).^9^^,^^10^

**Figure 1:**
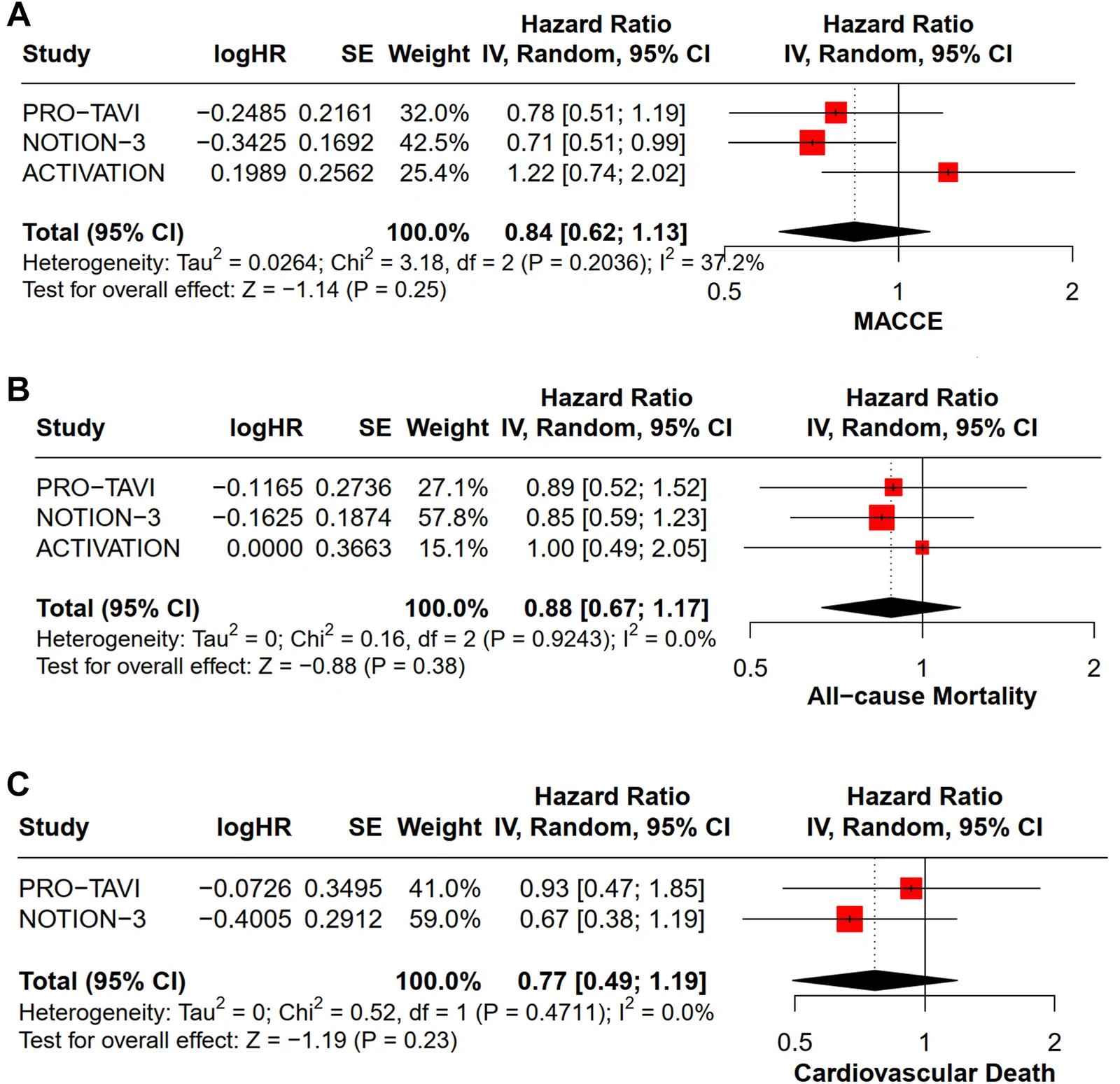
**Forest plotsof pooled hazard ratios.(A)** major adverse cardiovascular and cerebrovascular events (MACCE),^8^, ^9^, ^10^**(B)** all-cause mortality,^8^, ^9^, ^10^ and **(C)** cardiovascular death.^9^^,^^10^ HR, hazard ratio; SE, standard error.

### Pooled time-to-event analysis

Consistent with the primary study-level pooled data, this reconstructed patient-level analysis demonstrated no statistically significant difference in the risk of all-cause mortality between the 2 strategies (HR, 0.89; 95% CI, 0.68-1.17; *P* = .41). Over a follow-up period extending to 48 months, the cumulative incidence of all-cause mortality reached 36.3% in the PCI group compared with 46.2% in the deferral group (Figure 2). There was no violation of the PH assumption over the duration of follow-up (*P* = .38) (Supplemental Figure S3).

**Figure 2:**
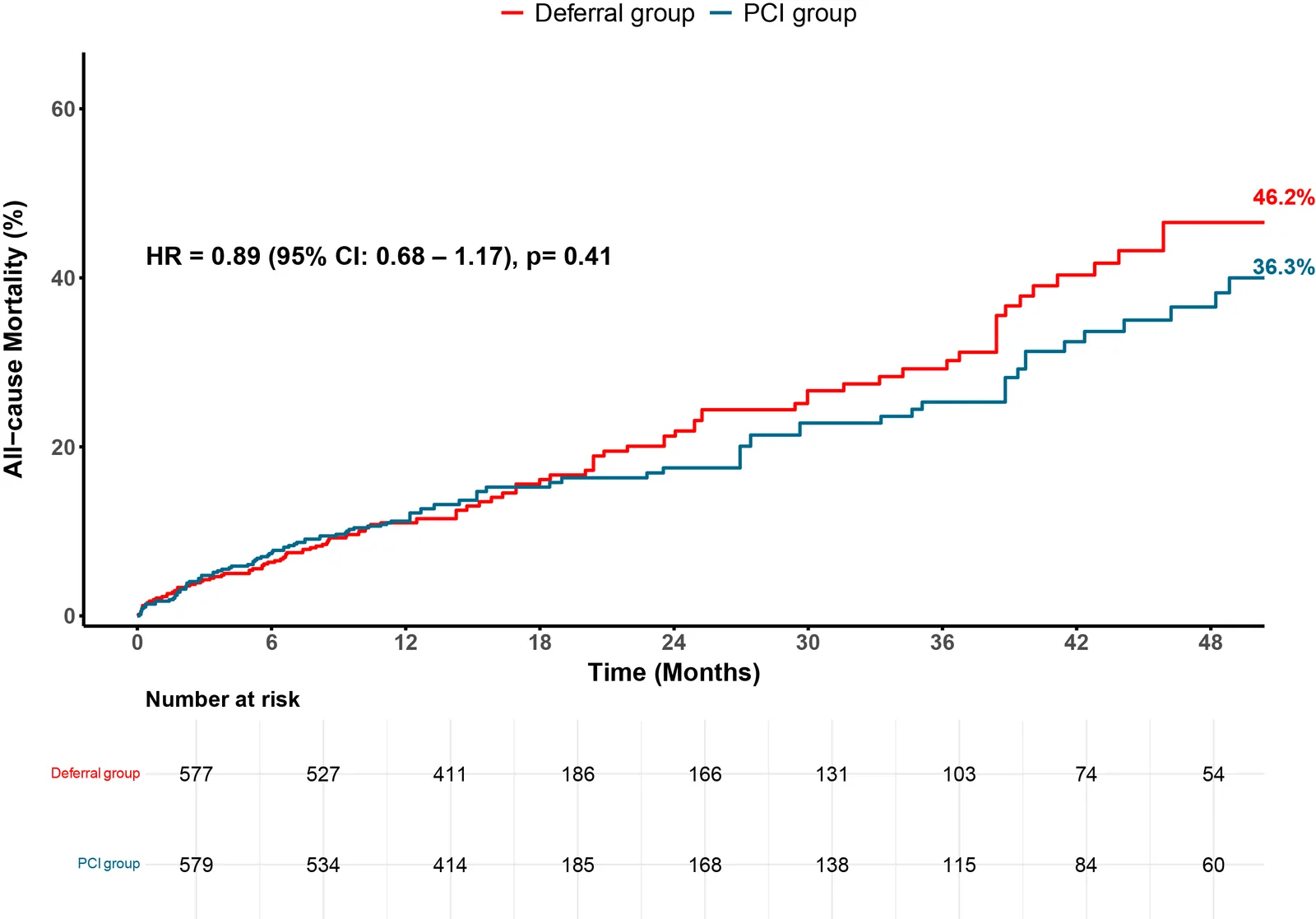
**Reconstructed Kaplan-Meier curves of pooled individual patient data for all-cause mortality.** HR, hazard ratio; PCI, percutaneous coronary intervention.

### Secondary ischemic outcomes

Percutaneous coronary intervention was associated with a significantly reduced risk of any revascularization (HR, 0.24; 95% CI, 0.06-0.89; *P* = .03; I^2^ = 82.3%) (Figure 3A)^9^^,^^10^ as well as urgent revascularization (HR, 0.33; 95% CI, 0.12-0.87; *P* = .02; I^2^ = 54.5%) (Figure 3B).^9^^,^^10^ Applying the pooled HR to the estimated 8.2% baseline risk in the deferral arm yields a number needed to treat of 19 to prevent 1 urgent revascularization event (Supplemental Table S4). Conversely, there was no significant difference between treatment strategies regarding the incidence of MI (HR, 0.84; 95% CI, 0.44-1.59; *P* = .59; I^2^ = 45.8%) (Figure 3C).^8^, ^9^, ^10^ Although there was a numerical trend toward a lower risk of stroke in the PCI arm, this finding did not reach statistical significance (HR, 0.69; 95% CI, 0.46-1.04; *P* = .07), with no observed heterogeneity across the 3 trials (I^2^ = 0.0%) (Figure 3D).^8^, ^9^, ^10^

**Figure 3:**
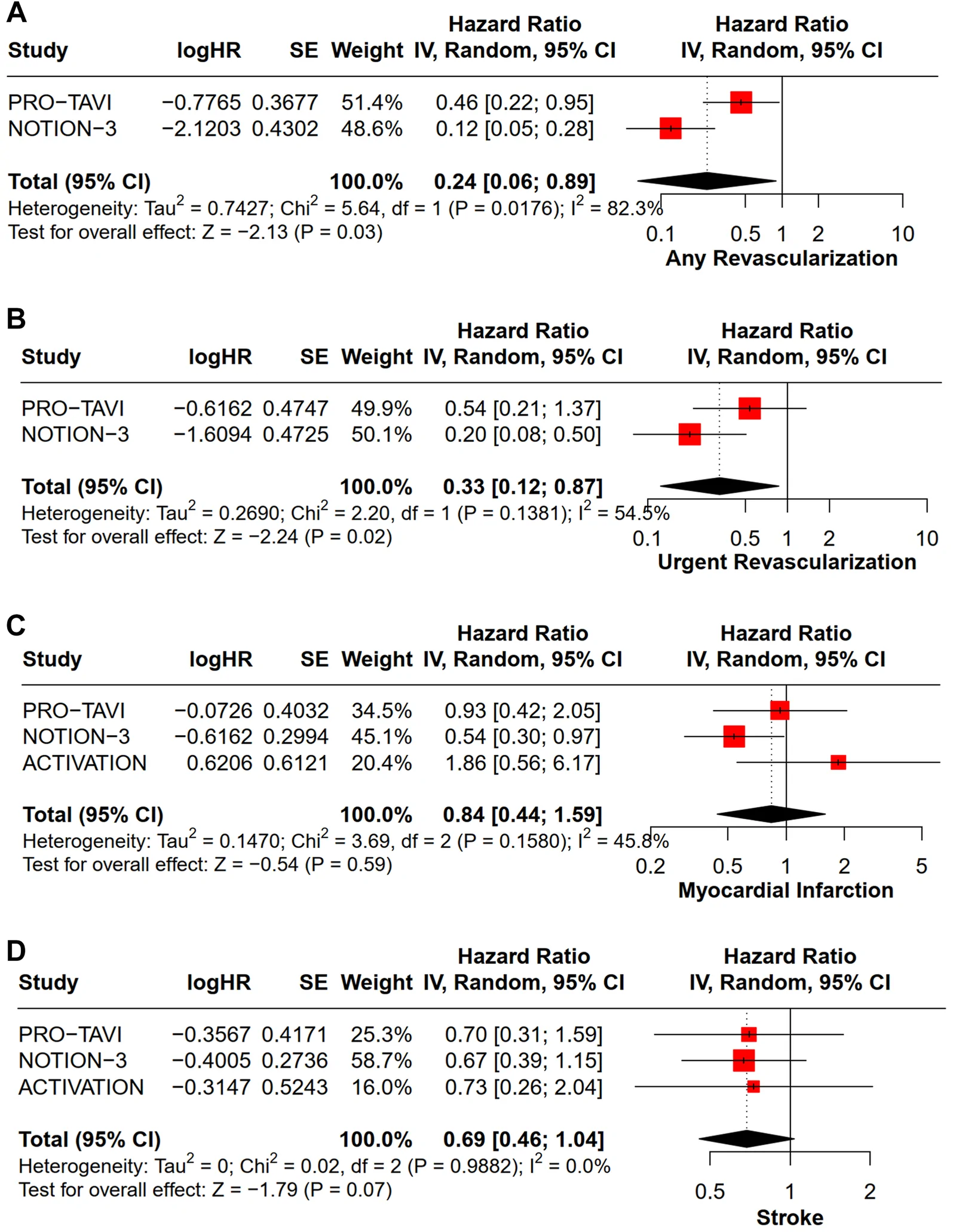
**Forest plots ofpooled hazard ratios.(A)** Any revascularization,^9^^,^^10^**(B)** urgent revascularization,^9^^,^^10^**(C)** myocardial infarction,^8^, ^9^, ^10^ and **(D)** stroke.^8^, ^9^, ^10^ HR, hazard ratio; SE, standard error.

### Safety outcomes

Analysis of safety end points revealed that PCI was associated with a significantly higher risk of any bleeding compared with the deferral group (HR, 1.96; 95% CI, 1.28-3.00; *P* < .01), with substantial between-study heterogeneity (I^2^ = 71.4%) (Supplemental Figure S4A), and a significantly higher risk of major bleeding compared with the deferral strategy (HR, 1.88; 95% CI, 1.07-3.31; *P* = .03), with moderate between-study heterogeneity (I^2^ = 45.4%) (Supplemental Figure S4B). Applying the pooled HR to the estimated 9.9% baseline risk in the deferral arm yields a number needed to harm of 13 for routine PCI to cause 1 additional major bleeding event (Supplemental Table S4). The incidence of acute kidney injury was comparable between the 2 arms, demonstrating no significant difference (HR, 1.20; 95% CI, 0.31-4.64; *P* = .79; I^2^ = 74.8%) (Supplemental Figure S4C).

### Sensitivity analyses

The sequential exclusion (leave-one-out analysis) of any single trial did not significantly alter the pooled effect estimates for any of the outcomes, with the exception of the MACCE outcome. It became statistically significant, favoring PCI following exclusion of the ACTIVATION trial (Supplemental Figures S3 and S5-S7).

## Discussion

This meta-analysis evaluated the optimal timing of PCI in patients undergoing TAVR. We found that routine PCI before valve implantation provides no survival benefit. Routine stenting significantly reduces the need for subsequent urgent revascularization. However, this ischemic benefit is associated with increased bleeding events (Central Illustration).^8^, ^9^, ^10^

**Central Illustration:**
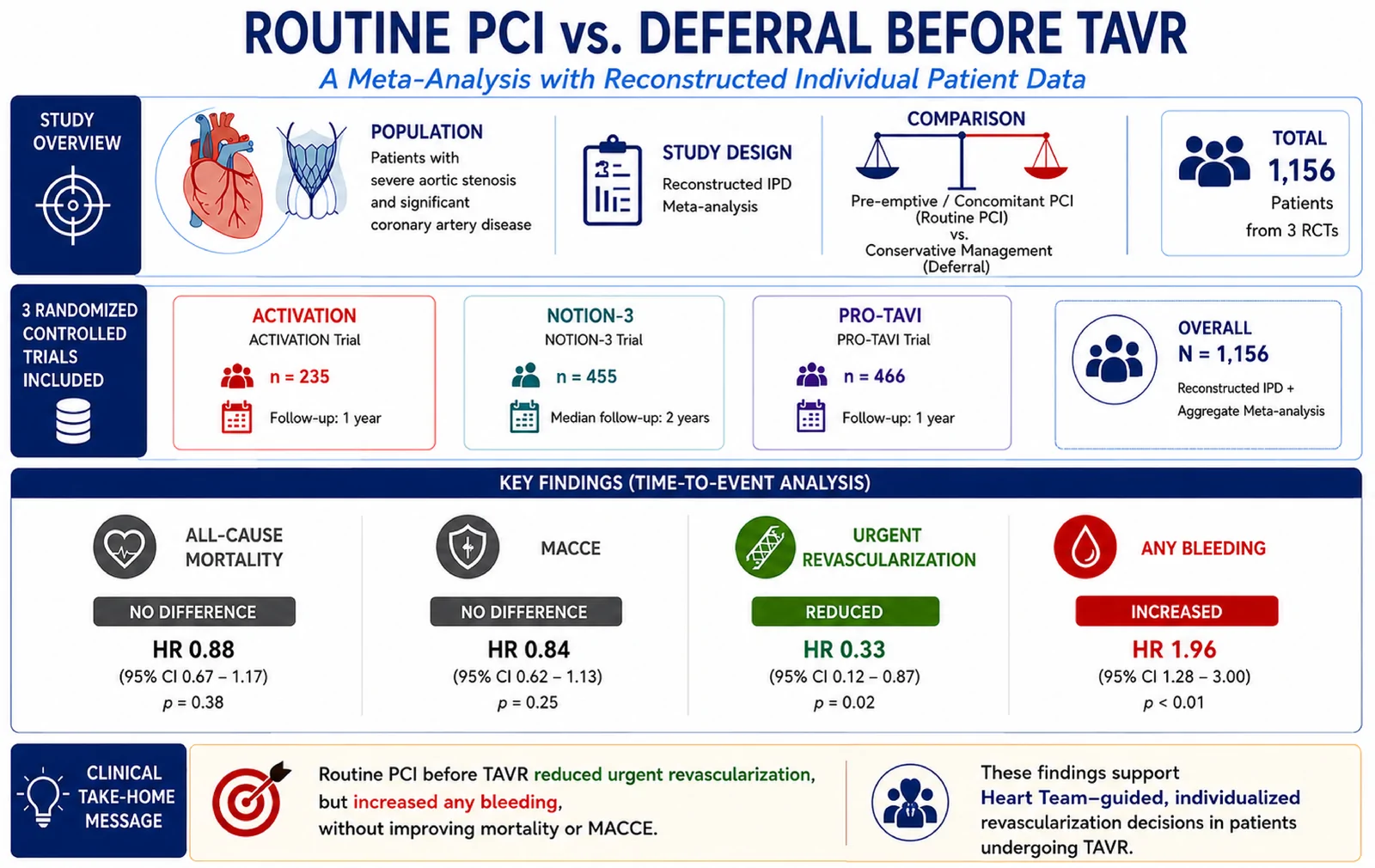
**Routine percutaneous coronary intervention (PCI) versus deferral in patients undergoing transcatheter aortic valve replacement (TAVR) with concomitant coronary artery disease.** A time-to-event meta-analysis of randomized controlled trials (RCT)^8^, ^9^, ^10^ comparing routine preemptive/concomitant PCI with a conservative deferral strategy in patients undergoing TAVR demonstrated no significant reduction in all-cause mortality or major adverse cardiovascular and cerebrovascular events (MACCE) with routine PCI. Although routine PCI decreased the need for subsequent urgent coronary revascularization, this benefit was accompanied by an increased risk of bleeding. HR, hazard ratio; IPD, individual patient data.

The absence of a mortality benefit aligns with the primary outcomes of the ACTIVATION and PRO-TAVI trials.^8^^,^^10^ Early observational registries previously suggested no clinical advantage for preemptive stenting.^15^ Our quantitative synthesis of purely randomized data confirms these observational signals. The relief of valvular obstruction may be the primary driver of survival in this demographic.

Routine PCI did show a reduction in the incidence of subsequent urgent revascularization. This finding agrees with the specific ischemic benefit reported in the NOTION-3 trial.^9^ However, the interpretation of this end point requires caution because it may be influenced by differences in physician decision-making. Patients assigned to the deferral strategy had documented, untreated coronary lesions. In clinical practice, awareness of this uncorrected anatomy introduces ascertainment bias; treating physicians likely maintain a lower threshold to perform urgent revascularization for subsequent anginal presentations compared to patients with known recent stenting. Such variability may partly explain the observed reduction in repeat revascularization without a corresponding reduction in mortality or MI.

We found no significant difference in the risk of spontaneous MI between the treatment strategies. This neutral effect may be clinically relevant. PCI did not significantly reduce infarction despite achieving anatomic revascularization. This finding is concordant with the PRO-TAVI trial, in which ischemic events were numerically higher after deferral but not significantly different.^10^ It also aligns with the broader stable ischemic heart disease literature, where routine revascularization was not found to reduce spontaneous MI in stable patients.^16^ The NOTION-3 trial reached a different conclusion. In that trial, PCI reduced the composite of death, MI, and urgent revascularization, driven primarily by fewer infarctions and repeat procedures.^9^ Design features may explain this divergence. The NOTION-3 trial used physiological assessment and treatment of FFR-positive lesions or angiographic stenoses of at least 90%, thereby enriching the population for truly ischemia-producing lesions.^9^ The ACTIVATION and PRO-TAVI trials relied predominantly on angiographic assessment.^8^^,^^10^ Physiological interrogation was limited in the PRO-TAVI trial and absent in most ACTIVATION trial participants.^10^ Severe AS alters coronary hemodynamics, complicating lesion assessment, and may dilute the benefit of revascularization. Our pooled results, therefore, may reflect real-world practice, where anatomic rather than physiological guidance remains the widely used strategy.^17^ Stroke showed a numerical reduction with PCI but did not reach statistical significance. Given the relatively low event rate, the analysis was likely underpowered. At present, the available evidence does not support routine PCI solely to reduce cerebrovascular events.

The safety analysis identified a profound hazard associated with routine coronary intervention. Prophylactic stenting nearly doubled the risk of bleeding compared to deferred management. This bleeding excess is primarily driven by the initiation of dual antiplatelet therapy in an older population.^6^ Intensified antithrombotic treatment amplifies hemorrhagic risk in an older population already susceptible to complications.^6^

Bleeding events after valve implantation are strongly associated with increased mortality.^18^^,^^19^ The reduction in late urgent revascularizations cannot justify the immediate bleeding risks imposed by mandatory dual antiplatelet therapy. A conservative strategy of deferring intervention appears highly durable in clinical practice. Most patients with stable coronary disease do not experience acute ischemic events following valve implantation.^20^

Our findings also align with prior observational syntheses. Lateef et al^15^ analyzed 11 cohort studies involving 5580 patients and found no reduction in 30-day mortality, stroke, MI, acute kidney injury, or 1-year mortality with PCI before or during TAVR. The present meta-analysis confirms those observations using randomized evidence, reducing confounding and selection bias inherent to observational studies.

The leave-one-out analysis offers additional insight. Excluding the ACTIVATION trial rendered the MACCE estimate statistically significant in favor of PCI. This may reflect the older TAVR era, smaller sample size, and early termination of the ACTIVATION trial.^8^ It also reflects the influence of the NOTION-3 trial, the largest and most contemporary trial, on ischemic end points.

Current guideline recommendations favor a selective approach.^1^^,^^7^ Our analysis supports that position. Coronary anatomy, lesion severity, symptom burden, bleeding risk, and anticipated post-TAVR coronary access should guide decision-making. When subsequent intervention is required, it can be performed safely.^21^ Commissural alignment strategies and newer transcatheter valve platforms increasingly facilitate coronary cannulation.^21^

Upcoming RCT will further delineate the optimal timing and anatomic indications for revascularization in this population. The pending TAVI PCI trial^22^ directly compares PCI before versus after valve deployment. Additionally, the large-scale COMPLETE TAVR trial^23^ will determine the efficacy of staged complete PCI versus isolated medical therapy following successful TAVR.

### Strengths and limitations

This meta-analysis represents the first synthesis of randomized evidence specifically evaluating routine PCI versus a conservative strategy in patients undergoing TAVR with concomitant CAD. We also reconstructed individual patient-level survival data over a follow-up period extending to 48 months.

Several limitations should be acknowledged. First, all included trials were open-label. This inherently risks ascertainment bias and may have influenced clinical decision-making regarding subsequent revascularizations. Second, the pooled cohort exhibits clinical heterogeneity regarding the definition of significant CAD. The ACTIVATION trial relied on an anatomic threshold of 70% diameter stenosis.^8^ The NOTION-3 and PRO-TAVI trials incorporated invasive physiological assessments.^9^^,^^10^

Anatomic ischemic burden also differed across studies. Left anterior descending artery stenosis was present in 61% of the NOTION-3 cohort. In contrast, the PRO-TAVI trial enrolled patients with only 21% involvement in this vessel, and only two-thirds of those lesions were proximal. This anatomic heterogeneity limits the generalizability of our ischemic end points and warrants future patient-level assessment. Additionally, the procedural timing of the intervention varied. The PRO-TAVI trial performed coronary intervention predominantly prior to valve implantation (93%). The NOTION-3 trial permitted concomitant and early postprocedural interventions. This procedural variability directly influences the incidence of periprocedural events.

The pooled MI end point incorporates varying trial definitions. The NOTION-3 trial included periprocedural infarctions occurring within 72 hours of valve implantation, thereby mixing iatrogenic biomarker elevations (type 4 and 5) with spontaneous ischemic events (type 1). This variance may complicate the interpretation of the isolated ischemic benefit. Furthermore, baseline oral anticoagulation use varied, reaching 36% in the PRO-TAVI cohort. Stroke etiology was not uniformly stratified into ischemic versus hemorrhagic subtypes. These data were incompletely reported across trials and could not be analyzed systematically.

Finally, our reconstructed time-to-event analysis extends to 48 months, but statistical power diminishes significantly beyond 18 months. Because the ACTIVATION and PRO-TAVI trials limited follow-up to 1 year, the late-term mortality data are driven predominantly by the NOTION-3 trial. Although this IPD reconstruction validates the PH assumption across the aggregate timeline, conclusions regarding mortality equivalence beyond 18 months require caution until extended follow-up data become available.

Our findings apply to patients with stable, low-complexity coronary anatomy. Patients with unprotected left main disease or acute coronary syndromes were systematically excluded. Deferring revascularization in these high-risk subsets remains clinically contraindicated. Because of the limited number of trials, we were unable to assess publication bias. In addition, subgroup analyses remain challenging in this population because of variability in coronary anatomy, ischemic burden, procedural timing, and antithrombotic strategies among trials. The limited randomized evidence base also restricted the ability to perform adequately powered subgroup analyses. Whether particular patient or lesion subsets derive differential benefit from routine PCI before TAVR remains uncertain and will likely need true individual patient-level data for reliable evaluation.

### Future recommendations

Future trials should standardize the assessment of coronary lesions in the setting of severe AS. Research should compare resting physiological indices against hyperemic FFR prior to valve deployment. We need trials dedicated specifically to patients with highly complex CAD. Evaluating deferred management in patients with SYNTAX scores exceeding 22 will clarify the absolute limits of a conservative strategy. Further investigation into optimal antithrombotic regimens is also necessary.

## Conclusion

This meta-analysis of RCT found that routine PCI before TAVR was not associated with a survival benefit. Routine PCI had the advantage of a small number of urgent revascularizations, however, with increased bleeding events. A conservative strategy of treating the aortic valve first and deferring coronary intervention should be considered safe and durable. Multidisciplinary heart teams should reserve preemptive coronary stenting for highly selected patients.

## CRediT authorship contribution statement

**Mahmoud Balata:** Writing – review & editing, Writing – original draft, Methodology, Data curation, Conceptualization. **Ahmed Samy Badran:** Writing – original draft, Methodology, Formal analysis, Data curation, Conceptualization. **Mohamed Ibrahim Gbreel:** Writing – original draft, Methodology, Data curation, Conceptualization. **Marwa Hassan:** Writing – original draft, Methodology, Conceptualization. **Islam Ebeid:** Writing – original draft, Methodology, Conceptualization. **Abdelhakim A. Abdelrahman:** Writing – original draft, Methodology, Data curation. **Daniel Robinson:** Writing – review & editing, Methodology, Conceptualization. **Jonas Bodanowitz:** Writing – review & editing, Methodology, Conceptualization. **Stephan Valenta:** Writing – review & editing, Methodology, Conceptualization. **Christin Valenta:** Writing – review & editing, Methodology, Conceptualization. **Plamen Kochev:** Writing – review & editing, Methodology, Conceptualization. **Hüseyin Ince:** Writing – review & editing, Methodology, Conceptualization. **Jasmin Ortak:** Writing – review & editing, Methodology, Conceptualization.

## Declaration of competing interest

The authors declared no potential conflicts of interest with respect to the research, authorship, and/or publication of this article.

## Footnotes

To access the supplementary material accompanying this article, visit the online version of the *Journal of the Society for Cardiovascular Angiography & Interventions* at 10.1016/j.jscai.2026.105566.
