# Supplementary material for: Routine Versus Deferred Percutaneous Coronary Intervention in Patients Undergoing Transcatheter Aortic Valve Replacement: A Meta-analysis With Reconstructed Individual Patient Data
Source: J Soc Cardiovasc Angiogr Interv. 2026 Aug 4;5(10):105566. doi: 10.1016/j.jscai.2026.105566 (PMC13700057; doi:10.1016/j.jscai.2026.105566)
Supplement: Supplementary file 1 [file mmc1.docx]

**Appendix**

- **Supplementary Tables**
  - **Table S1.** Search strategy for each database.
  - **Table S2.** Summary of the included studies.
  - **Table S3.** Trials definitions of MACCE outcome.
  - **Table S4.** Summary of Absolute Risk Metrics for Routine PCI versus Deferral
- **Supplementary Figures**
  - **Figure S1.** Flow diagram of study selection.
  - **Figure S2.** Risk of bias of the included studies using ROB-2 tool.
  - **Figure S3.** Testing for the Proportional Hazard assumption for the Kaplan-Meier curve in All-cause Mortality.
  - **Figure S4.** Forest plots of (A) Any Bleeding (B) Major Bleeding (C) Acute Kidney Injury.
  - **Figure S5.** Sensitivity analysis of (A) All-cause Mortality, (B) MACCE, (C) Cardiovascular Death
  - **Figure S6.** Sensitivity analysis of (A) Any Revascularization, (B) Urgent Revascularization, (C) Myocardial Infarction, (D) Stroke
  - **Figure S7.** Sensitivity analysis of (A) Any Bleeding (B) Acute Kidney Injury.

| Table S1. Search Strategy for each database. | | |
| --- | --- | --- |
| **Databases** | **strategy** | **No.** |
| **PubMed** | | |
| #1 | ( "Coronary Artery Disease"[Mesh] OR "Coronary Stenosis"[Mesh] OR "Percutaneous Coronary Intervention"[Mesh] OR "Angioplasty, Balloon, Coronary"[Mesh] OR coronary artery disease[tiab] OR coronary stenosis[tiab] OR coronary lesion*[tiab] OR coronary revascularization[tiab] OR coronary revascularisation[tiab] OR percutaneous coronary intervention*[tiab] OR coronary angioplasty[tiab] OR PCI[tiab] OR deferr*[tiab] OR conservativ* management[tiab] OR "no PCI"[tiab] OR "without PCI"[tiab] OR "no revascularization"[tiab] OR "no revascularisation"[tiab] OR routine PCI[tiab] OR complete revascularization[tiab] OR complete revascularisation[tiab] OR staged PCI[tiab] OR timing[tiab] OR sequence[tiab] ) | 1368601 |
| #2 | ( "Transcatheter Aortic Valve Replacement"[Mesh] OR "Aortic Valve Stenosis"[Mesh] OR "Heart Valve Prosthesis Implantation"[Mesh] OR "Transcatheter Aortic Valve Implantation"[tiab] OR "TAVI"[tiab] OR "Transcatheter Aortic Valve Replacement"[tiab] OR "TAVR"[tiab] OR "Percutaneous Aortic Valve Replacement"[tiab] OR "Percutaneous Aortic Valve Implantation"[tiab] OR "Catheter-based Aortic Valve Implantation"[tiab] OR "Catheter-based Aortic Valve Replacement"[tiab] OR "Aortic Valve Transcatheter Implantation"[tiab] OR "Aortic Valve Transcatheter Replacement"[tiab] OR "Edwards SAPIEN Valve"[tiab] OR "CoreValve"[tiab] OR "balloon-expandable valve"[tiab] OR "self-expanding valve"[tiab] ) | 87,426 |
| #3 | ("Randomized Controlled Trial"[pt] OR "Randomized Controlled Trials as Topic"[MeSH] OR "random*"[tiab] OR "RCT"[tiab] OR "randomised"[tiab] OR "randomized"[tiab] OR "randomly assigned"[tiab] OR "controlled trial*"[tiab]) | 1,970,349 |
| #4 | #1 AND #2 AND #3 | 420 |
| **Cochrane Library** | | |
| #1 | "Transcatheter Aortic Valve Implantation" OR "TAVI" OR "Transcatheter Aortic Valve Replacement" OR "TAVR" OR "Percutaneous Aortic Valve Replacement" OR "Percutaneous Aortic Valve Implantation" OR "Catheter-based Aortic Valve Implantation" OR "Catheter-based Aortic Valve Replacement" OR "Aortic Valve Transcatheter Implantation" OR "Aortic Valve Transcatheter Replacement" OR "Edwards SAPIEN Valve" OR "CoreValve" OR "balloon-expandable valve" OR "self-expanding valve" in Title Abstract Keyword | 1722 |
| #2 | "coronary artery disease" OR "coronary stenosis" OR "coronary lesion*" OR "coronary revascularization" OR "coronary revascularisation" OR "percutaneous coronary intervention*" OR "coronary angioplasty" OR PCI OR deferr* OR "conservativ* management" OR "no PCI" OR "without PCI" OR "no revascularization" OR "no revascularisation" OR "routine PCI" OR "complete revascularization" OR "complete revascularisation" OR "staged PCI" OR timing OR sequence in Title Abstract Keyword | 734880 |
| #3 | #1 AND #2 | 631 |
| **Scopus** | | |
| #1 | TITLE-ABS-KEY(  "Transcatheter Aortic Valve Implantation"  OR TAVI  OR "Transcatheter Aortic Valve Replacement"  OR TAVR  OR "Percutaneous Aortic Valve Replacement"  OR "Percutaneous Aortic Valve Implantation"  OR "Catheter-based Aortic Valve Implantation"  OR "Catheter-based Aortic Valve Replacement"  OR "Aortic Valve Transcatheter Implantation"  OR "Aortic Valve Transcatheter Replacement"  OR "Edwards SAPIEN Valve"  OR "CoreValve"  OR "balloon-expandable valve"  OR "self-expanding valve" ) |  |
| #2 | TITLE-ABS-KEY(  "coronary artery disease"  OR "coronary stenosis"  OR "coronary lesion*"  OR "coronary revascularization"  OR "coronary revascularisation"  OR "percutaneous coronary intervention*"  OR "coronary angioplasty"  OR PCI  OR deferr*  OR "conservativ* management"  OR "no PCI"  OR "without PCI"  OR "no revascularization"  OR "no revascularisation"  OR "routine PCI"  OR "complete revascularization"  OR "complete revascularisation"  OR "staged PCI"  OR timing  OR sequence ) |  |
| #3 | TITLE-ABS-KEY(  random* OR trial ) |  |
| #4 | #1 AND #2 AND #3 | 1,322 |
| **Web of Science (WOS)** | |  |
| #1 | TS=(  "Transcatheter Aortic Valve Implantation"  OR TAVI  OR "Transcatheter Aortic Valve Replacement"  OR TAVR  OR "Percutaneous Aortic Valve Replacement"  OR "Percutaneous Aortic Valve Implantation"  OR "Catheter-based Aortic Valve Implantation"  OR "Catheter-based Aortic Valve Replacement"  OR "Aortic Valve Transcatheter Implantation"  OR "Aortic Valve Transcatheter Replacement"  OR "Edwards SAPIEN Valve"  OR "CoreValve"  OR "balloon-expandable valve"  OR "self-expanding valve" ) |  |
| #2 | TS=(  "coronary artery disease"  OR "coronary stenosis"  OR "coronary lesion*"  OR "coronary revascularization"  OR "coronary revascularisation"  OR "percutaneous coronary intervention*"  OR "coronary angioplasty"  OR PCI  OR deferr*  OR "conservativ* management"  OR "no PCI"  OR "without PCI"  OR "no revascularization"  OR "no revascularisation"  OR "routine PCI"  OR "complete revascularization"  OR "complete revascularisation"  OR "staged PCI"  OR timing  OR sequence ) |  |
| #3 | TS=(  random* OR trial ) |  |
| #4 | #1 AND #2 AND #3 | 830 |

**Table S2.** Summary of the included studies

| **Study ID** | **Study Name** | **NCT** | **Design** | **Setting** | **Study Duration (Year)** | Total Sample size | **Definition of Significant CAD** | **Timing of PCI** | **Inclusion criteria** | **Follow-up (months)** | **Primary Outcome** |  |
| --- | --- | --- | --- | --- | --- | --- | --- | --- | --- | --- | --- | --- |
|  |  |  |  |  |  |  |  |  |  |  |  |  |
| Delewi 2026 | PRO-TAVI | NCT05078619 | Open-label, non-inferiority RCT | Multicenter (12 hospitals across the Netherlands) | 2021–2024 | 466 | 70–99% visual stenosis in a vessel ≥ 2.5 mm or bypass graft. Stenosis 40–70% included if FFR/iFR/RFR positive | concomitant | Indication for TAVI; clinically significant CAD (as defined) confirmed by Heart Team. | 12 | Composite of all-cause mortality, myocardial infarction, stroke, and major bleeding. |  |
| Lønborg 2024 | NOTION-3 | NCT03058627 | Open-label, superiority RCT | Multicenter (12 hospitals across Nordic-Baltic region) | 2017–2022 | 455 | FFR ≤ 0.80, or diameter stenosis ≥ 90% by visual assessment in a vessel ≥ 2.5 mm | Strongly recommended before TAVI (74% were done prior, 17% concomitant, 9% post) | Severe AS selected for TAVI; ≥1 physiologically or anatomically significant stenosis in a vessel ≥ 2.5 mm. | 24 (median) | MACE (composite of death from any cause, myocardial , or urgent revascularization). |  |
| Patterson 2021 | ACTIVATION | ISRCTN75836930* | Prospective, open-label, non-inferiority RCT | Multicenter (17 centers across UK, France, Germany) | 2012–2019 | 235 | ≥ 70% visual stenosis in a major epicardial artery (or ≥ 50% if protected left main/vein graft) | Prior to TAVR | Severe symptomatic AS suitable for TAVR; ≥ 1 stenosis ≥70% suitable for PCI; CCS angina class ≤ 2. | 12 | Composite of all-cause mortality or rehospitalization from any cause. |  |

| **Table S3.** Trial Definitions of MACE/MACCE Composite Outcomes | | | | |
| --- | --- | --- | --- | --- |
| **Study Name** | **Terminology Used** | **Outcome Classification** | **Composite Components** | **Follow-up Duration** |
| **ACTIVATION** | MACCE | Secondary Endpoint | Death, myocardial infarction, stroke, or acute kidney injury (AKI). | 30 days and 1 year |
| **NOTION-3** | MACE | Primary Endpoint | Death from any cause, myocardial infarction, or urgent revascularization. | Median 2 years (interquartile range, 1 to 4) |
| **PRO-TAVI** | Primary Composite | Primary Endpoint | All-cause mortality, myocardial infarction, stroke, or major bleeding. | 1 year |
|  | Secondary Composite | Secondary Endpoint | All-cause mortality, myocardial infarction, or stroke. | 1 year |
| MACCE = Major Adverse Cardiovascular and Cerebrovascular Events. MACE = Major Adverse Cardiac Events. * ACTIVATION defined its primary endpoint separately as a composite of all-cause mortality and rehospitalization at 1 year. * PRO-TAVI included a safety endpoint (major bleeding) within its primary efficacy composite.  *In the pooled analysis, we used the secondary Composite outcome * NOTION-3 defined urgent revascularization as any revascularization performed during an unplanned hospital admission for acute coronary syndrome. | | | | |

| **Table S4**. Summary of Absolute Risk Metrics for Routine PCI versus Deferral | | | | |
| --- | --- | --- | --- | --- |
| Clinical Outcome | Pooled Hazard Ratio (95% CI) | Pooled Baseline Risk (Control Event Rate; Deferral Arm) ᵃ | Absolute Risk Difference ᵇ | Number Needed to Treat / Harm ᶜ |
| Urgent Revascularization | 0.33 (0.12–0.87) | 0.082 | ARR: 5.4% | NNT = 19 |
| Major Bleeding | 1.88 (1.07–3.31) | 0.099 | ARI: 7.9% | NNH = 13 |
| ARI = Absolute Risk Increase; ARR = Absolute Risk Reduction; CI = Confidence Interval; NNH = Number Needed to Harm; NNT = Number Needed to Treat; PCI = Percutaneous Coronary Intervention. | | | | |
| ᵃ Pooled baseline risk (Control Event Rate) was calculated directly from the aggregate raw event counts in the deferral arms of the included trials reporting the specified outcome. | | | | |
| ᵇ Absolute risk differences were derived mathematically from the pooled hazard ratio and baseline risk using established formulas for time-to-event data (Altman & Andersen) [1]. | | | | |
| ᶜ Due to variable follow-up durations across the included trials (ranging from 12 months to a median of 24 months), the calculated NNT and NNH represent approximations over a blended 1-to-2-year temporal horizon. | | | | |
| Interpretation: For every 19 patients undergoing routine PCI before TAVI, one urgent revascularization is prevented. However, for every 13 patients undergoing routine PCI, one additional major bleeding event is caused. | | | | |

**[1]** Altman DG, Andersen PK. Calculating the number needed to treat for trials where the outcome is time to an event. BMJ. 1999. PMID: 10582940.

**Figure S1.** Flow diagram of study selection.

**Figure S2.** Risk of bias of the included studies using ROB-2 tool

**Figure S3.** Testing for the Proportional Hazard assumption for the Kaplan-Meier curve in All-cause Mortality.

**Figure S4.** Forest plots of (A) Any Bleeding (B) Acute Kidney Injury.

(A)

(B)

(C)

**Figure S5.** Sensitivity analysis of (A) MACCE, (B) All-cause Mortality, (C) Cardiovascular Death

**A)**

**B)**

**C)**

**Figure S6.** Sensitivity analysis of (A) Any Revascularization, (B) Urgent Revascularization, (C) Myocardial Infarction, (D) Stroke

**A)**

**B)**

**C)**

**D)**

**Figure S7.** Sensitivity analysis of (A) Any Bleeding (B) Major Bleeding (C) Acute Kidney Injury.

A)

B)

C)
